# The Influence of the Dominant Leg in Body Asymmetries in Children and Adolescent Male Soccer Players

**DOI:** 10.3390/pediatric16030058

**Published:** 2024-08-08

**Authors:** Eleni Theodorou, Theodoros B. Grivas, Marios Hadjicharalambous

**Affiliations:** 1 Human Performance Laboratory, Department of Life Sciences, University of Nicosia, 46 Makedonitissas Ave., P.O. Box 24005, Nicosia 1700, Cyprus; elenitheodorou@hotmail.com; 2 Department of Orthopedics & Traumatology, “Tzaneio” General Hospital of Piraeus, 18536 Piraeus, Greece; tgri69@otenet.gr

**Keywords:** leg dominance, training, postural asymmetry, functional scoliosis, youth soccer players

## Abstract

The current study aimed to examine (a) whether the dominant leg (DL) was associated with the contralateral side of functional scoliosis and (b) if any of the postural asymmetries’ evaluation variables may be a reliable predictor of the functional scoliosis development in young male soccer players. Six hundred-nine (*n* = 609) male soccer players (age: 10.8 ± 2.7 years; height: 147 ± 17 cm; weight: 43.4 ± 14.6 kg; DL: Right 81.6%, Left 14%, Both 4.4%) participated in this study. The spinal asymmetries evaluation included thoracic kyphosis, lumbar lordosis, truncal rotation, shoulders alignment from posterior view, anterior and posterior pelvic tilt, anterior superior iliac spine (ASIS), hamstring tightness, and lower extremities discrepancy. A significant association was observed between the DL and the truncal rotation side: χ^2^(4) = 30.84, *p* = 0.001, V = 0.16. Participants with longer left legs were likelier to present a spinal asymmetry (OR = 1.18). The participants with higher left shoulders were 2.13 times more likely to have spinal asymmetry than the participants with normal shoulders level. Participants with left ASIS higher were 3.08 times more likely to present asymmetry than those with normally aligned ASIS levels. There was also a significant association between the DL and the side of truncal rotation: χ^2^(2) = 13.30, *p* = 0.001, V = 0.449. Logistic regression analysis for the functional scoliotic group and truncal rotation side demonstrated that the taller participants and participants with shorter right legs were more likely to have asymmetry on the left side (OR = 1.29, OR = 0.32). Participants with greater right hamstring stiffness were likelier to have a truncal rotation on the right side (OR = 0.93). Participants with higher left shoulders were 0.20 times less likely to have a truncal rotation on the left side than the participants with normal shoulders level. In conclusion, leg dominance in children and in youth soccer players may be a factor causing truncal rotation on the contralateral side. Additional causes, such as leg length discrepancy and pelvic tilt, may progressively lead to functional scoliosis.

## 1. Introduction

Correct body posture has been defined as the position where minimum stress is applied to each joint, while incorrect posture is any static position that increases stress at the joints [1]. Incorrect body posture would result in severe deterioration of scoliosis with age [2,3,4,5] since in developmental ages, an early and abrupt body growth may result in an unequal growth of the various body’s structures, which may subsequently lead to changes in the balance of the musculoskeletal system and skeletal alignment, particularly and progressively to functional scoliosis [1,2,3,5,6,7,8]. Functional scoliosis is characterized by the disturbance of the spinal balance in orthostatism without experiencing any anatomical deterioration of the vertebrae or any influence of the intervertebral disks [9]. Several genetic and epigenetic factors, such as environmental (i.e., nutrition, smoking, alcohol, medication, obesity, toxins, and viruses), biochemical, and neurological, are considered major causes of scoliosis development [8,10].

In sport settings, it was recently found that the skeletal maturation rate associated with early sports specialization may signify risk factors for particular injury types in adolescent athletes [11], and skill development through regular strenuous practice may affect muscle balance and skeletal alignment [6]. It has been reported, for example, that sports involving throwing and kicking patterns that demand predominantly unilateral execution may negatively influence muscle imbalance [6,12,13,14,15]. In soccer, for example, a high degree of laterality-induced postural asymmetries and muscle imbalance is exhibited due to functional long-term specific training adaptations [16,17,18,19,20]. Elite adolescents and young soccer players (14–20 years of age) present substantial functional deficits, in deep squat and trunk stability tasks particularly, and asymmetry between the left and right sides [21]. It has been recently also observed that kyphotic and scoliotic postural asymmetries negatively contribute to deteriorating neuromuscular explosiveness performance and diminishing lower limbs’ flexibility in young international-level soccer players [22]. However, an 8-week individual corrective-specific exercise intervention program contributed to improving postural and musculoskeletal asymmetry status in young male soccer players [17]. Consequently, the scoliotic curvature might be corrected by using specific exercises, which include exercises that place the body in various corrective positions [9,17,23]. If functional scoliosis is not corrected, it may progressively develop structural scoliosis, which will predispose the soccer players to injuries or decrease their exercise performance [2,23].

It was also presumed that upper-body postural deformities occur as a result of the player’s body position when they are passing the ball during daily practice [7,19]. The majority of the soccer players have a preferable dominant leg, which is a prominent factor for developing asymmetry in muscular strength and in flexibility of the lower extremities [7]. It was found, for example, that the laterality in certain sports is correlated with asymmetrical adaptations in bones and muscle circumference, hips’ flexibility, and muscular strength [24,25]. Furthermore, in young athletes, any chronic anatomical adaptation developed due to the laterality of sport may affect their future athletic performance, predisposing them to injuries and negatively influencing their future quality of life [2,7,26]. For example, in adolescents, it was found that regular basketball and volleyball training may negatively affect body posture, increasing progressively the deviation of body posture, while Olympic gymnast training demonstrated symmetrical body posture development [27].

However, further research is required for examining whether regular soccer training may negatively affect adolescents’ body posture [27]. Such examination will reveal valuable information to develop focused testing and intervention strategies in an attempt to reduce as much as possible inter-limb asymmetries [14]. To the authors’ knowledge, no studies so far have been conducted to examine the relationship between leg dominance and postural asymmetries in adolescents soccer players. Therefore, the aim of the current study was to examine whether the dominant leg (DL) was associated with the contralateral side of truncal rotation and whether any of the postural asymmetries’ evaluation variables may be a reliable predictor of the development of functional scoliosis in youth male soccer players. It was hypothesized that DL may affect the development of functional scoliosis on the contralateral side. 

## 2. Materials and Methods

### 2.1. Participants

The targeted sample for this study was 800 young male soccer players. Initially, the researcher contacted the private academies association and the National Sports Development Support Plan Commission. Both bodies voluntarily informed the soccer academies of th9s study and sought their participation. The academies that accepted the invitation to participate in this study have sent the prepared envelopes by the researchers, including the relevant study information, to the parents. The final number of participants in the main study were six hundred-nine (*n* = 609) male soccer players (age: 10.8 ± 2.7 years; height: 147 ± 17 cm; weight: 43.8 ± 15 kg; dominant leg (DL): Right 81.6%, Left 14%, Both 4.4%), all active members of soccer academies. This study was approved by the National Bioethics Committee (ΕΕΒΚ/ΕP/2017/39) and conformed with the Code of Ethics of the World Medical Association (Declaration of Helsinki). Written informed consent was obtained by the players and their parents prior to any assessments and after explaining the experimental methods and procedures. Participants who were injured or sick during the assessment period were excluded from this study.

### 2.2. Experimental Design

The assessments took place in the fitness centers of the soccer academies. The coaches of the teams and the parents were allowed to be present during all testing procedures. In cases where severe postural asymmetries were found, an assessment report was sent, enclosed in an envelope, to the parents or guardians at the end of the evaluation, recommending a visit to an orthopedic specialist. 

### 2.3. Postural and Muscular Asymmetries Evaluations

Postural malalignments were assessed according to Kendall [2]. Thoracic kyphosis, lumbar lordosis, truncal rotation at any level of the spine, shoulders alignment from posterior view, pelvic tilt (anterior or posterior), anterior superior iliac spine level, hamstring tightness, and lower extremities discrepancy test were evaluated. Thoracic kyphosis and lumbar lordosis were evaluated using an inclinometer (AcuAngle^®^ Inclinometer, Baseline Evaluation Instruments, Los Angeles, CA, USA), and the truncal rotation was evaluated with a scoliometer (Mizuho Osi^®^, Mizuho OSI Inc., Tokyo, Japan). Using a goniometer (Baseline 12-100HR, HiRes^®^ Goniometer w/360° Head-12” Arm-25/Pack, Los Angeles, CA, USA), the assessment of the hamstring tightness (90-90 test) and the pelvic tilt have been performed. 

#### 2.3.1. Assessment of Thoracic Kyphosis and Lumbar Lordosis

Thoracic kyphosis was assessed by measuring the angle formed during the spinous processes of the first and second thoracic (T1/T2) and the twelfth thoracic and first lumbar vertebrae (T12/L1). In a standing position, the participant bent his head forward smoothly to identify the T1/T2. Thus, the spinous process of the seventh cervical vertebra (C7) could be identified. Then, the researcher placed the inclinometer on the T1/T2 spinous processes for obtaining the first measurement for thoracic kyphosis. The inclinometer was then placed on the T12/L1 spinous processes in order to obtain the second measurement. Then, the examiner, by following the last rib to the spine, palpated the T12. From that point, the researcher also palpated the L1 spinous process, and finally, the thoracic kyphosis angle was calculated by subtracting the first measurement from the second [28,29,30]. Then, the lumbar lordosis was assessed by measuring the angle formed by the spinous processes of T12/L1 and the spinous processes of the second sacral vertebra (S2) and third sacral vertebra (S3). S2 is located in the center of the two posterior superior iliac spines, and the S3 is below. The measurements of T12/T1 during thoracic kyphosis assessment and the measurement for the S2/S3 were obtained. Next, the inclinometer was placed on S2/S3 to evaluate the spinous processes. Finally, the lumbar lordosis was calculated by subtracting the second measurement from the first to have a positive number. The thoracic kyphosis and lumbar lordosis angles were estimated by using the linear and triangular addition of the angles rule, as previously described [28,29,30].

#### 2.3.2. Assessment of Truncal Rotation (Thoracic, Thoracolumbar, and Lumbar)

The participants initially performed Adam’s forward bending test with extended arms touching their knees for thoracic and thoracolumbar measurements, closing their feet. During Adam’s bending forward test, the researcher clinically observed whether there was an asymmetry (i.e., truncal that is hemithoracic) at any spine level, placing the scoliometer at that particular level for obtaining the degree of ATR (Angle of Trunk Rotation) scoliosis and the convexity side of truncal rotation [31].

#### 2.3.3. Shoulder Alignment

The shoulders level was assessed by observing from a posterior side and having the participant normally standing, recording whether there was a shoulder malalignment indicating the higher side [6].

#### 2.3.4. Pelvic Tilt

Using a goniometer, the pelvic tilt was assessed by measuring the angle formed of imaginary lines connecting the anterior superior iliac spine (ASIS) and the posterior superior iliac spine (PSIS). The participant initially stood, and the researcher palpated the ASIS and PSIS. Then, the examiner placed one goniometer arm on an imaginary line connecting the ASIS and PSIS and the other arm parallel to the floor. Next, the angle in degrees between the two arms of the goniometer was recorded, assessing whether the pelvis was within normal limits or tilted anteriorly or posteriorly [1].

#### 2.3.5. Anterior Superior Iliac Spine (ASIS) Level Examination

The participant stood with straight legs, and then the researcher palpated ASIS bilaterally to assess whether the ASIS were aligned. In the case of malalignment, the higher side of ASIS was reported. The evaluation of ASIS was based on the anterior plumb line passing through the body’s midsagittal plane [1,6].

#### 2.3.6. Hamstring Tightness

The active knee extension test or 90-90 test was used to assess the hamstring tightness. The participant should lay in a supine position on the examination bed. Then, the researcher positioned the hip and knee in flexion at 90 degrees of the tested leg and kept the ankle in plantar flexion. Next, the contralateral leg was held straight and down to the bed (90-90 test). Finally, the researcher maintained the hip in 90° flexion, and the participant extended his knee to the limit of motion where hamstring muscles were in full stretch, obtaining the measurement by determining the knee angle produced by the midline of the thigh and distal fibula using a universal goniometer [32]. 

#### 2.3.7. Leg Length

The leg length was measured using a meter tape while the participant was on the examination bed. Initially, the examiner aligned the pelvis and measured the distance from the anterior superior iliac spine to the medial malleolus, which measures the actual leg length [1].

### 2.4. Statistical Analysis

A priory pilot study (*n* = 29 male soccer players; age: 9.1 ± 0.9 years old, height: 137 ± 9 cm, weight: 34 ± 9 kg) tested the intra-tester reliability of the inclinometer and scoliometer. Intraclass Correlation Coefficient (ICC), 95% Confidence Interval (CI), and *p*-value were reported. Following the test of the normality of distribution (Kolmogorov–Smirnov), all data were reported as the median and interquartile range (IQR). Since all data violated the assumptions for parametric analysis, a non-parametric evaluation was performed using the Chi-square test (χ^2^) to examine the study’s hypothesis. The Cramer’s V value was also requested to identify the effect size of the association of the tested variables. According to Cohen (1988), Cramer’s V value of 0.1 is considered a small effect size, 0.3 represents a medium effect size, and 0.5 represents a large effect size [33]. Logistic regression analysis was performed to identify whether any of the variables that had been measured were reliable predictors of the development of functional scoliosis. For all the logistic regression analyses stepwise, the Backward LR method was used due to the exploratory nature of the investigation. 

## 3. Results

### 3.1. Intraclass Correlation Coefficient

The results for the intra-tester reliability of the inclinometer and scoliometer demonstrated excellent reproducibility (Table 1).

### 3.2. Demographics

Table 2 presents the median and the interquartile range (IQR) of truncal rotation degrees of the whole sample and the scoliosis group.

**Figure 1:**
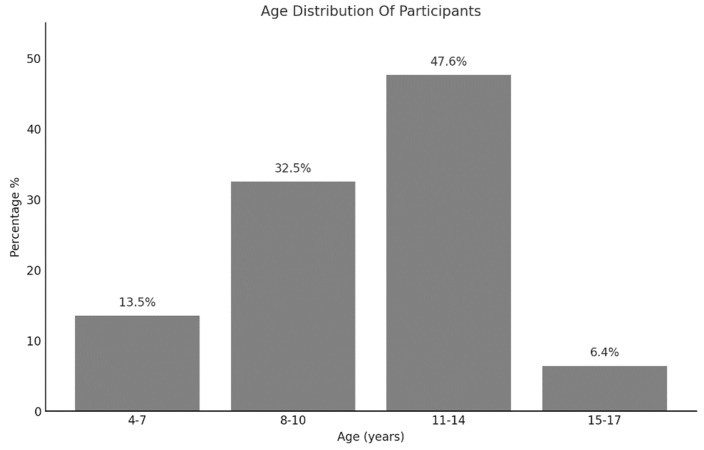
Age distribution of the participants.

Participants were categorized according to their age. The frequency and the percentage of each category are presented in Figure 1.

### 3.3. Chi-Square (χ^2^)—Association of the Dominant Leg and Truncal Rotation Side

There was a significant association between the dominant leg (DL) and the truncal rotation side: χ^2^(4) = 30.84, *p* = 0.001, V = 0.16. The effect size for this finding, Cramer’s V, was small. Within the functional scoliotic posture group (five degrees and above) [31], there was also a significant association between the DL and the side of truncal rotation: χ^2^(2) = 13.30, *p* = 0.001, V = 0.449.

### 3.4. Logistic Regressions—Whole Sample

The first logistic regression model with the truncal rotation degrees as the outcome and the scale variables as the predictors resulted in a statistically significant model, χ^2^(3) = 19.09, *p* < 0.001, with two significant predictors: standing height and left leg length, using the whole sample. The model explained the 6.6% (Nagelkerke R^2^) of the truncal rotation degree variance and correctly classified 89.6% of the participants (Table 3).

The second logistic regression model with the truncal rotation degree as the outcome and the categorical variables as the predictors resulted in a statistically significant model, χ^2^(4) = 19.91, *p* = 0.001, with two significant predictors: higher shoulder side (posterior view) and higher ASIS side, using the whole sample. Thus, the model explained 6.8% (Nagelkerke R^2^) of the truncal rotation variance and correctly classified 89.5% of the participants (Table 4).

### 3.5. Logistic Regressions—Functional Scoliosis Group

The first logistic regression model with the truncal rotation side as the outcome and the scale variables as the predictors resulted in a statistically significant model, χ^2^(5) = 12.96 *p* < 0.05, with three significant predictors: standing height, right leg length, and right hamstring stiffness, using the functional scoliosis group. The model explained 26.4% (Nagelkerke R^2^) of the truncal rotation variance and correctly classified 70.5% of the participants (Table 5).

The second logistic regression model with the truncal rotation side as the outcome and the categorical variables as the predictors resulted in a statistically significant model, χ^2^(4) = 11.42, *p* < 0.05, with two significant predictors: pelvic tilt classification and higher shoulder side (posterior view), using the functional scoliosis group. The model explained 22.3% (Nagelkerke R^2^) of the variance in the truncal rotation side and correctly classified 72.7% of the participants (Table 6).

## 4. Discussion

The primary aim of the current study was to assess the potential association between the dominant leg and the contralateral side of truncal rotation. The secondary aim of the experiment was to examine whether any of the evaluated independent variables regarding body posture could be a valid predictor for identifying the progress of scoliosis in young male soccer players between the ages of 4 and 17 years old. 

### 4.1. Leg Dominance

The main findings demonstrated a significant association between the dominant leg (DL) and the truncal rotation side (χ^2^(4) = 30.84, *p* = 0.001), with a small effect size (V = 0.16). Within the scoliotic posture group (5 degrees and above) [34], there was also a significant association between the DL and the side of truncal rotation (χ^2^(2) = 13.30, *p* = 0.001), with a large effect size (V = 0.449). Most participants presented truncal rotation on the contralateral side of the DL. Based on scoliosis categorization, 10.8% (*n* = 66) of the participants fell in the scoliotic group [35]. Considering the solid characteristic asymmetrical nature of soccer [16] and the fact that most soccer players have a dominant leg, this preference can result in strength and flexibility asymmetry of the legs [2]. A relationship between asymmetrical movements of the lower body due to sports training requirements and pelvic asymmetry was found in elite athletes participating in unilateral sports starting training during their pre-pubertal age. Possibly all the adaptations made due to the laterality of sport, which may affect their future performance, predispose them to injuries, and impact their future quality of life as adults [2,24]. It was also found that the lateral dominance of an athlete may also influence the pelvic asymmetry observed in unilateral athletes [2,22]. In asymmetric sports athletes, fatigue had a greater impact on the DL, and the DL had a greater influence on postural control. It was suggested that both fatigue and the nature of the sport could influence postural control, with possibly more considerable effect on asymmetric sports athletes [36]. In our study, the revealed cross laterality of the DL and side of truncal rotation and the pelvis tilt [as functional scoliosis predictor] seem to be correlated with the leg preference; therefore, soccer laterality can affect children and young soccer players’ posture. 

Several studies demonstrated that the laterality in certain sports could correlate with asymmetrical adaptations in bones and muscle circumference and muscles’ flexibility and strength [24,25]. Taketomi et al. reported musculoskeletal asymmetries between the dominant and non-dominant legs in young male and female soccer players in muscle strength, balance, and flexibility. Notably, the DL was significantly stronger in knee flexion and hip abduction in males, with a higher hamstring/quadriceps ratio and more flexible quadriceps than the non-dominant leg [37]. Unilateral overload may also result in deviations and functional changes in the spine and joints [2,38,39]. 

Using the DL in soccer, unilateral load results and deviations in the measured parameters, such as truncal rotation, pelvic asymmetry, and hamstring flexibility imbalance, were found in our study. Soccer laterality is different compared to other sports and may cause further muscle imbalances or deviations. Hanimann et al. [16] found that the direction of laterality differed between young soccer players and alpine skiers. Soccer players showed larger medial knee displacement during drop jump landings on the non-dominant side and larger dead-bug bridging displacement on the dominant side, while skiers showed the opposite pattern. Lateralities should be analyzed based on sport-specific thresholds and consider the potential functional advantages and disadvantages of individual asymmetries in athletes [16]. In a recent school scoliosis screening study, cross laterality was found only in schoolboys within the general sample and the scoliotic group, between the dominant hand and leg with the truncal rotation side [40]. However, in the aforementioned and in the current study, schoolboys were not categorized as athletes and non-athletes. Future comparisons between young male soccer players and young male non-athletes would reveal whether this cross-laterality is found only in soccer players or the general young boys’ population. 

### 4.2. Predictors for Functional Scoliosis in Youth Soccer

Based on the logistic regression analysis, taller participants were less likely to have asymmetry for the whole sample and truncal rotation degrees as the outcome (OR = 0.93). Although the mean height of the participants with a normal spine was slightly lower (m = 147 cm) than those with an asymmetric spine (m = 152 cm), the height of slightly taller participants with a normal spine seems to have led to this finding. Also, participants with longer left legs were more likely to present asymmetry (OR = 1.18). Participants with higher left shoulders were 2.13 times more likely to have spinal asymmetry than the participants with normal shoulder levels. Also, participants with a higher left ASIS were 3.08 times more likely to present asymmetry than those with normally aligned ASIS levels. However, based on the logistic regression analysis for the scoliotic group and truncal rotation side as the outcome, the taller participants and participants with shorter right legs (therefore, longer left legs) were more likely to have asymmetry on the left side (OR = 1.29 and OR = 0.32). Also, participants with greater right hamstring stiffness were more likely to have a truncal rotation on the right side (OR = 0.93). The participants with anterior pelvic tilt were 4.3 times more likely to present truncal rotation on the left side than those with a normally aligned pelvis. Also, participants with higher left shoulders were 0.2 times less likely to have a truncal rotation on the left side than those with normal shoulders level. 

Functional scoliosis occurs more frequently than structural scoliosis [41]. There is an agreement that functional scoliosis is an asymmetry in the coronal plane without evidence of a thoracic hump or lumbar asymmetry based on Adam’s test [42]. It could be noticed by either the spine, prominence of the ribs, or asymmetry of the pelvis and shoulders level [43]. Various factors may cause functional scoliosis, such as leg length inequality, hip dysplasia, pelvic obliquity, muscular imbalances, poor posture, joint issues, and upper extremities asymmetry [9,42,43,44,45]. For example, it was previously found that international U15 and U17 soccer players with scoliotic postures had lower sit-and-reach flexibility scores compared to those with normal postures. This suggests that scoliotic posture may reduce hamstring flexibility [22]. In the current study, participants with greater right hamstring stiffness were likelier to have a truncal rotation on the right side. Thus, it implies that hamstring stiffness coexists in scoliotic posture. Similarly, in our study, shoulders level, leg length inequality, and ASIS level (pelvis tilt) seem to be predictors for functional scoliosis. Leg length discrepancy is a frequent orthopedic condition found in approximately 10% of children [46,47], which results in pelvic tilt in the frontal plane [26,42,45,46,47] and functional scoliosis [26,42,45,46,47]. 

In addition, leg length discrepancy may cause structural changes in the spine over time [26,46]. In our study, participants with more than five degrees of truncal rotation possibly had leg length inequality for a long time. Grivas et al. [48] found that leg length inequality was correlated with pelvic rotation, pelvic tilt, and surface rotation. The angle of trunk rotation at the lumbar level was also correlated to the pelvic tilt. Leg length inequality was also associated with sagittal imbalance and trunk inclination in degrees. No correlation was found between the leg length inequality and structural scoliosis angle [48]. According to Marsiolo et al. [42], for every millimeter of leg length inequality, there is a predicted 0.12 degrees of vertebral rotation; as the leg length inequality increases, the likelihood of vertebral rotation also increases. However, it is important to note that this correlation is specific to functional scoliosis caused by leg length inequality and may not apply to other types of scoliosis [42]. In several cases, functional scoliosis may cause further asymmetries with its onset. It may also result in abnormal postural habits, altered biomechanics, muscular imbalances, joint laxity, and excessive strain on the spine’s joints, muscles, tendons, and bones [23,26]. It is important to note that functional scoliosis is different from structural scoliosis and can often be improved or resolved by addressing the underlying cause [42]. 

Due to continuous asymmetrical loadings, professional soccer players may present functional and structural adaptations of the neuromusculoskeletal system. Hajduk and Schmidtbleicher [49] found that thoracolumbar functional scoliosis and ASIS level were associated with side-specific adaptations of young elite soccer players between 16 and 19 years old. Specifically, the U19 players were found to be associated with lumbar scoliosis. Researchers concluded that the functional postural adaptations were more complex as players aged [49]. The abovementioned parameters may alter the spinal posture, predispose to injuries, and negatively affect young soccer players’ performance. Therefore, future steps should be taken considering the study’s novel finding, the association of the dominant leg with the contralateral side of truncal rotation, and the association with the body height, pelvis tilt, shoulders level, leg length inequality, and hamstring stiffness. 

### 4.3. Limitations

There was no laterality assessment, and the players and their coaches reported the dominant leg. The cut-off values of the collected measured variables were based on adults’ values reported in the literature and not on children’s and adolescent’s values, except for truncal rotation degree measurements. 

One significant limitation of our study is the absence of sport-specific testing measurements, particularly those that assess change of direction (COD) and linear sprint abilities. Therefore, the ability to detect and understand the relationship between change of direction, linear sprint abilities, and observed postural asymmetries is limited. Incorporating these tests into future studies would provide a more comprehensive understanding of how postural asymmetries affect athletic performance and help in developing targeted interventions to mitigate any negative impacts.

The lack of maturity status assessment in this study is another limitation, as it is well-established that maturity status can impact physical performance, particularly in young athletes, as indicated by prior research [50,51,52,53].

Since screening was performed, no radiography was used. It is important to consider that functional (nonstructural) scoliosis might progress to structural scoliosis; therefore, a longitudinal follow-up study will reveal significant information on how functional scoliosis might progress in youth soccer players. 

There was no control group (non-athlete boys) to compare these findings since this study’s main goal was to examine whether there was an association between the young male soccer players. Future comparisons between young male soccer players and young male non-athletes should be conducted.

## 5. Conclusions

The present study provides valuable insights into the relationship between leg dominance and the development of functional scoliosis in youth male soccer players, independently of the four age categories evaluated in the current study. A significant association was found between the dominant leg and the contralateral side of truncal rotation, indicating that leg dominance may contribute to the development of spinal asymmetries. Additionally, this study identified several postural asymmetries, such as leg length discrepancy, pelvic tilt, and shoulder height, as potential predictors of functional scoliosis. Participants with longer left legs, higher left shoulders, and higher left ASIS were more likely to present with spinal asymmetry. Moreover, taller participants and those with shorter right legs were more prone to left-sided truncal rotation, while greater right hamstring stiffness was associated with right-sided truncal rotation. These findings underscore the importance of considering leg dominance and other postural factors in the early detection and prevention of functional scoliosis among kids and youth athletes. Further research is warranted to explore the underlying mechanisms and to develop targeted intervention strategies for the mitigation of scoliosis risks in this population.

## Figures and Tables

**Table 1:** Intraclass Correlation Coefficient.

	Intraclass Correlation Coefficient (ICC)	95% Confidence Interval (CI)	*p*-Value
Scoliometer	0.988	0.981–0.993	0.001 *
Inclinometer T1-T2	0.987	0.979–0.993	0.001 *
Inclinometer T12-L1	0.995	0.991–0.997	0.001 *
Inclinometer S2-S3	0.995	0.991–0.997	0.001 *

Spinous process of: T1 = first thoracic vertebra; T2 = second thoracic vertebra; T12 = twelfth thoracic vertebra; L1 = first lumbar vertebra; S2 = second sacral vertebra; and S3 = third sacral vertebra. * Statistically significant difference.

**Table 2:** Truncal rotation degrees.

	Truncal Rotation Degrees	
	N	Median ± IQR
Whole Group	609	2 ± 2
Functional Scoliosis Group	66	5 ± 1

**Table 3:** A logistic regression model with truncal rotation degrees as the outcome and the scale variables as predictors—whole sample.

Variable	B (SE)	Odds Ratio	95% CI for Odds Ratio	
			Lower	Upper
Standing Height	−0.07 (0.03) *	0.93	0.87	1
Kyphosis	−0.02 (0.01)	0.98	0.95	1
Left Leg Length	0.16 (0.06) **	1.18	1.06	1.32
Constant	−4.10 (1.42) **	0.02		

*, **: Indicates statistically significant predictor at the 0.05 level and 0.01 respectively, for truncal rotation degrees for the whole sample.

**Table 4:** A logistic regression model with truncal rotation degrees as the outcome and the categorical variables as predictors—whole sample.

Variable	B (SE)	Odds Ratio	95% CI for Odds Ratio	
			Lower	Upper
Higher Shoulder Side (posterior view)				
Right (v. none)	0.17 (0.34)	1.18	0.6	2.32
Left (v. none)	0.75 (0.33) *	2.13	1.11	4.08
Higher ASIS Side				
Right (v. none)	0.47 (0.58)	1.61	0.52	4.97
Left (v. none)	1.12 (0.31) **	3.08	1.67	5.68
Constant	−2.62 (0.21) **	0.07		

*, **: Indicates statistically significant predictor at the level 0.05 and 0.01 respectively, for the truncal rotation degrees for the whole sample.

**Table 5:** A logistic regression model with the truncal rotation side as the outcome and the scale variables as predictors—functional scoliosis group.

Variable	B (SE)	Odds Ratio	95% CI for Odds Ratio	
			Lower	Upper
Standing Height	0.25 (0.12) *	1.29	1.02	1.62
Sitting Height	−0.24 (0.14)	0.79	0.6	1.04
Right Leg Length	−1.15 (0.55) *	0.32	0.11	0.93
Left Leg Length	0.93 (0.52)	2.53	0.92	6.98
Right Hamstrings Stiffness	−0.07 (0.03) **	0.93	0.89	0.98
Constant	0.77 (3.38)	2.16		

*, **: Indicates statistically significant predictor at the 0.05 level and 0.01 respectively for truncal rotation degrees for the whole sample.

**Table 6:** A logistic regression model with the truncal rotation side as the outcome and the categorical variables as predictors—functional scoliosis group.

Variable	B(SE)	Odds Ratio	95% CI for Odds Ratio	
			Lower	Upper
Pelvic Tilt Classification				
Anterior (v. normal)	1.46 (0.65) *	4.3	1.19	15.46
Posterior (v. normal)	−0.09 (0.91)	0.92	0.16	5.41
Higher Shoulder Side (posterior view)				
Right (v. none)	−0.87 (0.75)	0.42	0.1	1.82
Left (v. none)	−1.63 (0.69) *	0.2	0.05	0.75
Constant	0.99 (0.53) *	2.68		

*: Indicates statistically significant (*p* < 0.05) predictor for truncal rotation side.

## Data Availability

The data presented in this study are available upon request from the corresponding author.
